# 454. Impact of Covid-19 on Infectious Disease Fellows in the United States: A National Survey to Identify Targets for Intervention

**DOI:** 10.1093/ofid/ofab466.653

**Published:** 2021-12-04

**Authors:** Shuba Balan, Shweta Anjan, Alison Ohringer, Jose Gonzales-Zamora, Deborah Jones Weiss, Michele I Morris, Maria L Alcaide, Paola Lichtenberger, Paola Lichtenberger

**Affiliations:** 1 Department of Infectious Diseases, University of Miami, Miami, Florida; 2 University of Miami / Jackson Memorial Hospital, Miami, Florida; 3 University of Miami/Jackson Health System, Miami, Florida; 4 University of Miami, Miller School of Medicine, Miami, Florida; 5 University of Miami Miller School of Medicine, Miami, Florida; 6 University of Miami MIller School of Medicine, Miami, FL

## Abstract

**Background:**

The Coronavirus disease of 2019 (COVID-19) global health crisis has resulted in an unprecedented strain on healthcare systems, reorganization of medical training programs and disruption in professional and personal lives of medical trainees. The impact of COVID-19 on infectious disease (ID) fellows, who are frontline healthcare professionals, has not been assessed.

**Methods:**

We conducted a national survey of adult and pediatric ID fellows to assess impact on educational activities, availability of personal protective equipment (PPE), well-being, and career prospects. Anxiety and burnout were assessed by 7-item generalized anxiety disorder scale and abbreviated Maslach burnout inventory respectively. Invitations to participate in the survey were sent via email to all ID fellows through Accreditation Council for Graduate Medical Education (ACGME) fellowship directors. Survey responses collected from August 1 to September 30, 2020 have been reported.

**Results:**

136 fellows completed the survey (Table 1). 84% reported their institution had provided evidence-based didactics for management of COVID-19 and 53% indicated their general ID didactics were affected by the pandemic. 86% of fellows were involved in care of patients with COVID-19, and 31% reported a shortage of PPE affecting their clinical duties. Those living in highly impacted states (CA, FL, NY, TX) at the time of the survey were 1.70 times as likely to experience moderate to severe anxiety (vs. minimal to moderate) than those in other states; similarly, those who saw ≥11 COVID-19 patients weekly and reported PPE shortages were 2.5 and 2.0 times as likely, respectively, to experience moderate to severe anxiety compared to their peers who took care of 10 or fewer COVID-19 patients and did not experience PPE shortages. Burnout scores were not significant (Table 2).

Table 1. Demographics, Responses to Personal Exposure, Educational Activities and Career Prospects

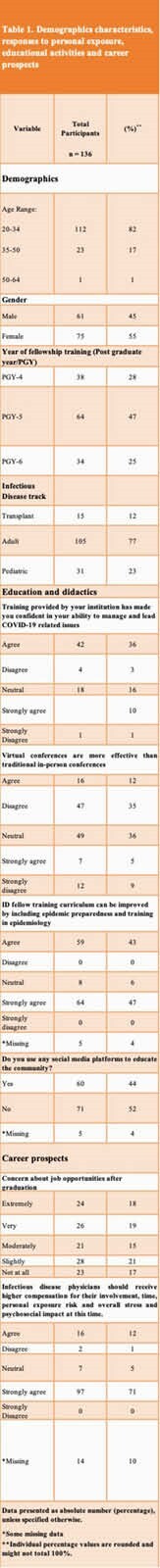

Table 2. Stress, Burnout, Anxiety , Sleep and Quality of Life Among Survey Participants.

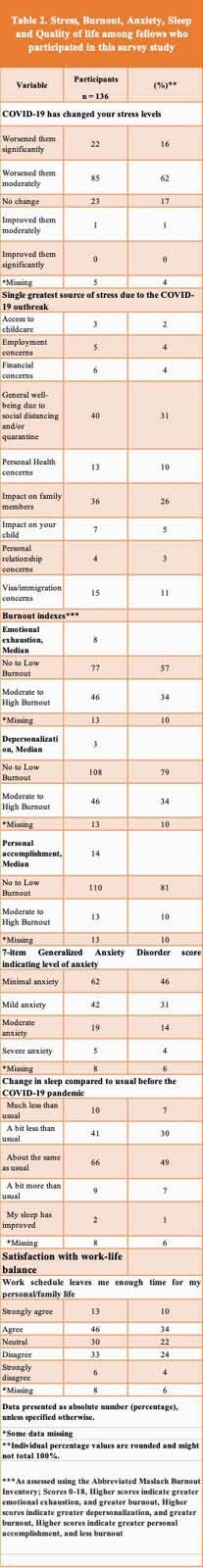

**Conclusion:**

It is imperative that ID fellows feel adequately protected and supported during this pandemic. Pandemic preparedness should be included in the ID fellowship curriculum. Interventions for anxiety and burnout reduction should be implemented. ID fellowship programs should continue to accept feedback from fellows to ensure their ongoing safety, well-being, and education as we navigate this pandemic.

**Disclosures:**

**All Authors**: No reported disclosures

